# Single-cell transcriptome sequencing reveals tumor heterogeneity in family neuroblastoma

**DOI:** 10.3389/fimmu.2023.1197773

**Published:** 2023-09-18

**Authors:** Yunlong Zhang, Yue Ma, Qingqing Liu, Yifei Du, Liang Peng, Jianwu Zhou, Zhenzhen Zhao, Changchun Li, Shan Wang

**Affiliations:** Department of Pediatric Surgical Oncology Children’s Hospital of Chongqing Medical University, National Clinical Research Center for Child Health and Disorders, Ministry of Education Key Laboratory of Child Development and Disorders, Chongqing Key Laboratory of Pediatrics, Chongqing, China

**Keywords:** family neuroblastoma (FNB), single-cell RNA sequencing (scRNA-seq), single-cell flux estimation analysis (scFEA), SDHD, cancer-associated fibroblasts (CAF), tumor microenvironment

## Abstract

Neuroblastoma(NB) is the most common extracranial solid tumor in childhood, and it is now believed that some patients with NB have an underlying genetic susceptibility, which may be one of the reasons for the multiplicity of NB patients within a family line. Even within the same family, the samples show great variation and can present as ganglioneuroblastoma or even benign ganglioneuroma. The genomics of NB is still unclear and more in-depth studies are needed to reveal its key components. We first performed single-cell RNA sequencing(sc-RNAseq) analysis on clinical specimens of two family neuroblastoma(FNB) and four sporadic NB cases. A complete transcriptional profile of FNB was constructed from 18,394 cells from FNB, and we found that *SDHD* may be genetically associated with FNB and identified a prognostic related CAF subtype in FNB: Fib-4. Single-cell flux estimation analysis (scFEA) results showed that malignant cells were associated with arginine spermine, oxaloacetate and hypoxanthine, and that malignant cells metabolize lactate at lower levels than T cells. Our study provides new resources and ideas for the development of the genomics of family NB, and the mechanisms of cell-to-cell interactions and communication and the metabolic landscape will provide new therapeutic targets.

## 1 Introduction

Neuroblastoma (NB) is a heterogeneous solid tumor that arises in the sympathetic nervous system (1). The main clinical feature of NB is the heterogeneity of its tumor, and the possibility of cure varies greatly depending on the age at diagnosis, the extent of the disease, and the biology of the tumor (2). The vast majority of NB occurs sporadically, and approximately 1-2% of neuroblastoma cases are inherited within families (3). It is currently believed that the genetic susceptibility to NB will follow an autosomal dominant pattern of inheritance with an epistasis of approximately 63% (4). The two-hit hypothesis is considered the most consistent model of inherited predisposition to NB (5).In addition, even FNB occurring in the same family line can show considerable heterogeneity, including ganglioneuroblastoma and even benign ganglioneuroma (6, 7). In most cases of FNB, there are no specific associated clinical features in these cases. However, a small proportion of patients with NB have clinically identifiable genetic syndromes associated with NB, including congenital central hypoventilation syndrome (CCHS), aganglionosis of the colon (Hirschsprung disease), ROHHAD syndrome (rapid-onset obesity, hypothalamic dysfunction, hypoventilation, and autonomic dysfunction), and neurofibromatosis type 1, all of which are characterized by neural crest developmental disorders.

The gene *ALK *(8–10), paired-like homeobox 2B (*PHOX2B*) (11, 12), has been found to be strongly associated with FNB by techniques such as Genome-wide linkage analysis, and there are also case reports that *GALNT14* may be associated with genetic susceptibility to NB (13). A previous study suggested that the 2p (D2S162-2S2259) and 12p (D12S1725-D12S1596) regions are novel locations for FNB susceptibility genes (14). However, there are still some FNB that do not exhibit the specific genomic alterations described above. The genetics of FNB is still only partially understood and continued research is expected to reveal new insights into FNB susceptibility, including gene-gene and gene-tumor microenvironment (TME) interactions. In recent years, single-cell RNA sequencing (scRNA-seq) analysis has made rapid progress in the study of NB by using individual cells as resolution such as NB has a predominant chromaffin-cell-like phenotype (15), malignant tumor cells can differentiate into fibroblasts (16) and so on. This also gives us a new tool to study FNB. Here, we analyzed two cases of FNB from two separate families by scRNA-seq analysis in an attempt to investigate potential genetically related sites, detect the genetic pattern of the tumor, and further reveal the genomic features of FNB, while providing a solid foundation for studying the development of NB.

## 2 Case description

These two FNB were from two different family lines. At the time of F1’s diagnosis, her brother had already died from multiple metastases of NB throughout his body.F2 was the younger one of a pair of twins. F2’s sister was found to have a tumor in the adrenal region, while no obvious tumor was found during the regular follow-up in pregnancy (including prenatal B ultrasound and MRI) before they were born. At the age of seven months after birth, F2 went to our hospital for abdominal pain, and no primary tumor site was found in all examinations. Neuroblastoma was only found in the liver and was confirmed by biopsy pathology.

In the beginning, the author suspected that the liver lesions of F2 were metastasis tumors because of transplacental transmission, which was also consistent with the anatomical basis of single chorionic double amniotic sac placenta (17), but we lacked the results of the biopsy of placental tissue. To further prove the source of the tumor, immunohistochemistry was used. There were significant differences in the results of Ki-67 (**Supplementary Figure 1A, C**) and S-100 (**Supplementary Figures 1B, D**) expression levels between F2 and F2’s sister (**Supplementary Figures 1A, B** belong to F2; **Supplementary Figures 1C, D** belong to F2’s sister).Immunohistochemistry showed that the F2 tumor was more indolent, and the tumor cell density, mitosis-karyorrhexis index(MKI), and mitotic index of F2 were all lower than her sister’s tumors. Therefore, we thought that the tumor of F2 is not metastatic, because the metastatic focus should usually be more invasive. Moreover, these two tumors have completely different Shimada histology (**Table 1**), and the diagnosis age of F2 and her sister was less than 12 months (18). As reported, genetic factors predominate in neuroblastoma diagnosed in newborns and infants (19), so we believed that F2 was a familial neuroblastoma without a primary site.

**Table 1:** Clinical information of six samples including two FNB.

Characteristics	F1	F2	A8	A53	A5	A24
Age of diagnosis	3-y-old girl	27-d-old girl	3-y-old girl	20-m-old girl	2-m-old girl	4-m-old boy
clinical diagnosis	ganglionneuroblastoma	Hepatic metastasis of neuroblastoma	neuroblastoma	neuroblastoma	neuroblastoma	neuroblastoma
Family history	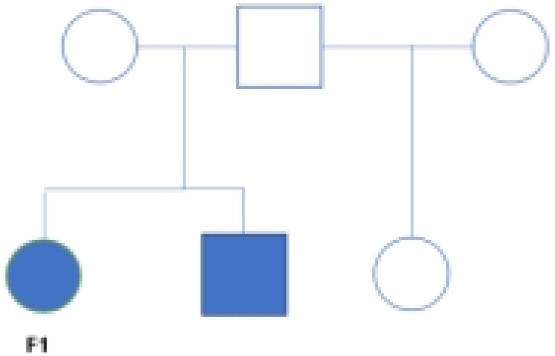	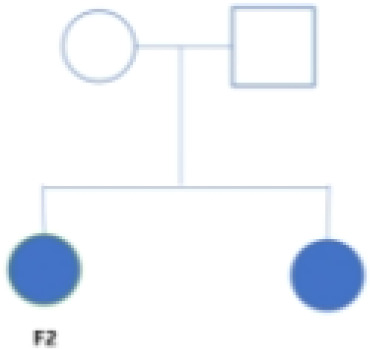	/	/	/	/
Primary site	Left retroperitoneum	No primary site	Right retroperitoneum	Right retroperitoneum	Left retroperitoneum	Left retroperitoneum
Shimada histology	uFH	FH	uFH	uFH	FH	FH
*N-MYC*	no amplification	no amplification	no amplification	no amplification	no amplification	no amplification
MKI	/	low	medium	/	medium	medium
INRG	L1	MS	L2	L1	MS	MS
Status at last follow-up	Alive	Alive	Alive	Alive	Alive	Alive

## 3 Results

### 3.1 Cellular diversity in family neuroblastoma

**Figure 1:**
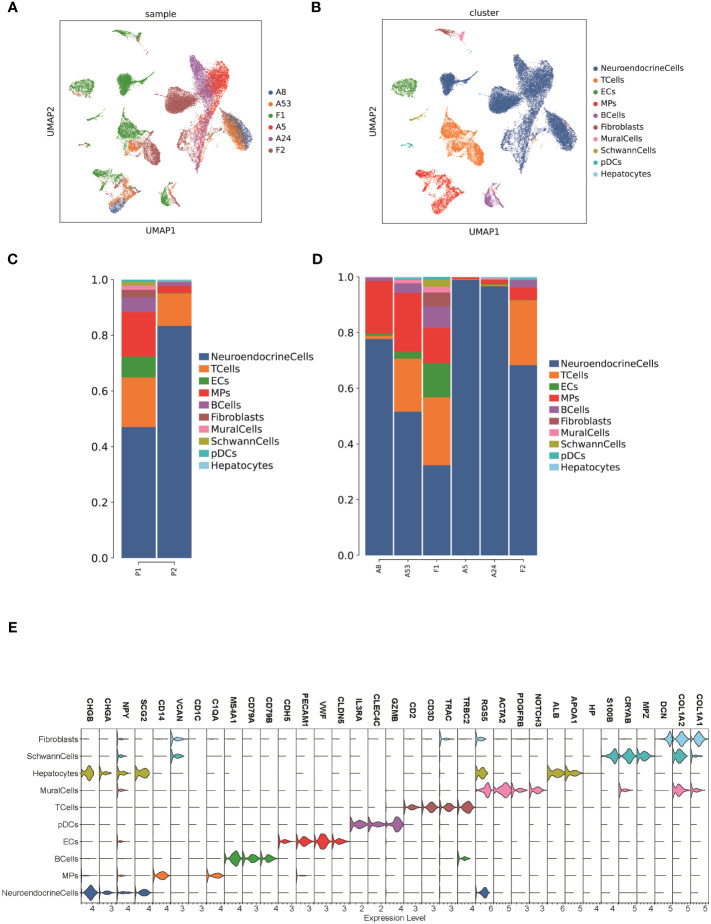
The TME of family neuroblastoma and sporadic neuroblastoma tissues. **(A)** The UMAP plot of 6 samples. **(B)** The UMAP plot of each cell type in 6 samples. **(C)** Histogram of the relative proportions of each cell type for the 2 groups. **(D)** Histogram of the relative proportions of each cell type for the 6 samples. **(E)** Violin plots of the expression of marker genes in each cell type.

To investigate the tumor heterogeneity of FNB, we sequenced a total of six samples (**Figure 1A**) including two cases of FNB(F1, F2). The six samples were divided into two groups. The P1 group included F1 and two cases of stage I/II sporadic neuroblastoma (A8 and A53), while the P2 group included F2 and two cases of stage IVs sporadic neuroblastoma (A5 and A24) (**Figure 1C**). All six samples were examined histopathologically, including one case of ganglioneuroblastoma and five cases of neuroblastoma, and *N-MYC* was not amplified in all cases. After stringent quality filtering, we obtained a total of 35,369 cells from the six samples, and two cases of familial neuroblastoma were identified with 9482 and 8912 cells. A total of ten cell types were identified in the six samples, including neuroendocrine cells(NEs), Schwann cells, T cells, B cells, mononuclear phagocytes(MPs), fibroblasts, endothelial cells(ECs),mural cells, plasmacytoid dendritic cells(pDCs), and hepatocytes (**Figure 1B**). The cell types shared by the six samples included neuroendocrine cells (NEs), T cells, ECs, and MPs. Uniform manifold approximation and projection (UMAP) was used to visualize cell clusters and violin plots were used to show the canonical markers in each cell type (**Figure 1E**). Consistent with the literature, the T cells were distinguished by high expression of CD2,CD3D,TRAC, and TRBC2 (20, 21), while the B cells were characterized by high expression of MS4A1,CD79A, and CD79B (20, 22). The pDCs specifically expressed IL3RA, CLEC4C, and GZMB (23, 24); the MPs had high expression of CD14, VCAN, CD1C, and C1QA (20, 25, 26); and the mural cells had high expression of RGS5,ACTA2,PDGFRB, and NOTCH3 (27, 28).We also identified fibroblasts by high expression of DCN,COL1A2, and COL1A1 (29, 30), ECs by high expression of CDH5,PECAM1,VWF, and CLDN5 (31), and NEs by high expression of CHGB,CHGA,NPY, and SCG2 (32, 33).The schwann cells were characterized by the expression of S100B,CRYAB,and MPZ (34, 35), the hepatocytes were characterized by the expression of ALB,APOA1,HP (26, 36).

We further compared the proportion of cell types in FNB versus sporadic neuroblastoma, and among the six shared cell types, we found that FNB had fewer NE cells and more immune cells in terms of number share (**Figure 1D**), which also represented a more complex tumor immune microenvironment and closer immune cell-to-cell communication in FNB compared to sporadic neuroblastoma. In addition, we identified partial hepatocytes in F2, associated with tissue samples that could carry partial liver tissue.

### 3.2 Identification of malignant cells in FNB

**Figure 2:**
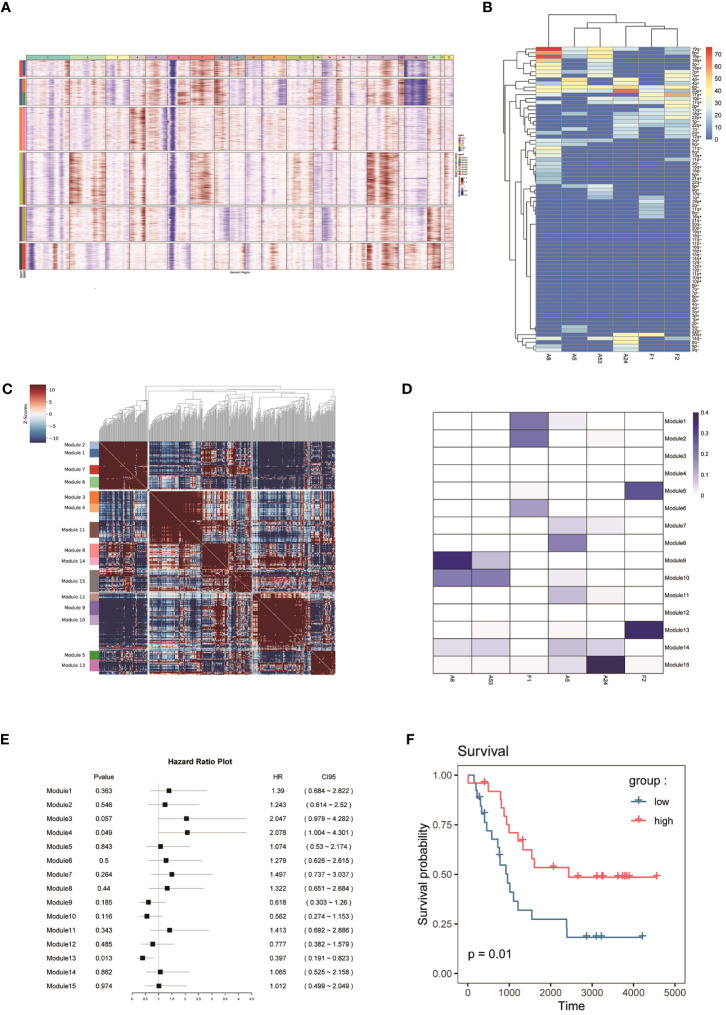
**(A, B)** CNV analysis of NEs in 6 samples. **(C)** Hotspot analysis of NEs in 6 samples. **(D)** Jaccard similarity analysis of NEs in 6 samples based on the hotspot analysis. **(E)** Survival analysis of 15 modules. **(F)** Kaplan–Meier(KM) survival curve of the module 13.

To classify the cells into malignant and non-malignant cells, we used the inferred CNV algorithm (**Figure 2A**) to demonstrate the clonal structure of the cells. The results showed that NEs have more CNVs than other cell types. By comparison, we observed significant chromosome 17p gain in two cases of FNB and only chromosome 19p gain in F1 (**Figure 2B**). Similarly, we also focused on mesenchymal cells including fibroblasts and endothelial cells, and the results showed that they have few CNVs, so we considered NEs to be malignant cells.

### 3.3 SDHD probably associated with genetic susceptibility to FNB

To study the genomic features of NEs in FNB, we performed a Hotspot analysis (**Figure 2C**) of NEs (37). We obtained a total of 15 modules, and by Jaccard similarity analysis (**Figure 2D**), we performed one-to-one correspondence between gene modules and tumor samples. We found that A8 and A53, A5 and A24 had a similar module expression, but no more consistent module expression was observed between F1 and F2, where F1 mainly expressed module1 (including *RPS2, RPL18A, RPS29, RPL39*), module2 (including *RPL34, RPS27, MTND1P23, RACK1*), module6 (including *MT-CO2, MTATP6P1, RPL35, MT-CO3*), F2 mainly expressed in module5 (including *MEG3, JUN, HSPA1A, FOS*), and module13 (including *CALM2, TMSB10, TUBA1A, TMSB4X*). Then, we performed survival analysis of the 15 modules (**Figure 2E**) and we found that the module 13 in highly expressed F2 was associated with better survival. The module13 included genes currently known to be associated with poor prognosis (**Figure 2F**) including *MIF* (38), *DDX5* (39), and also genes associated with good prognosis including *UCHL1* (40), and *STMN4* (41).

**Figure 3:**
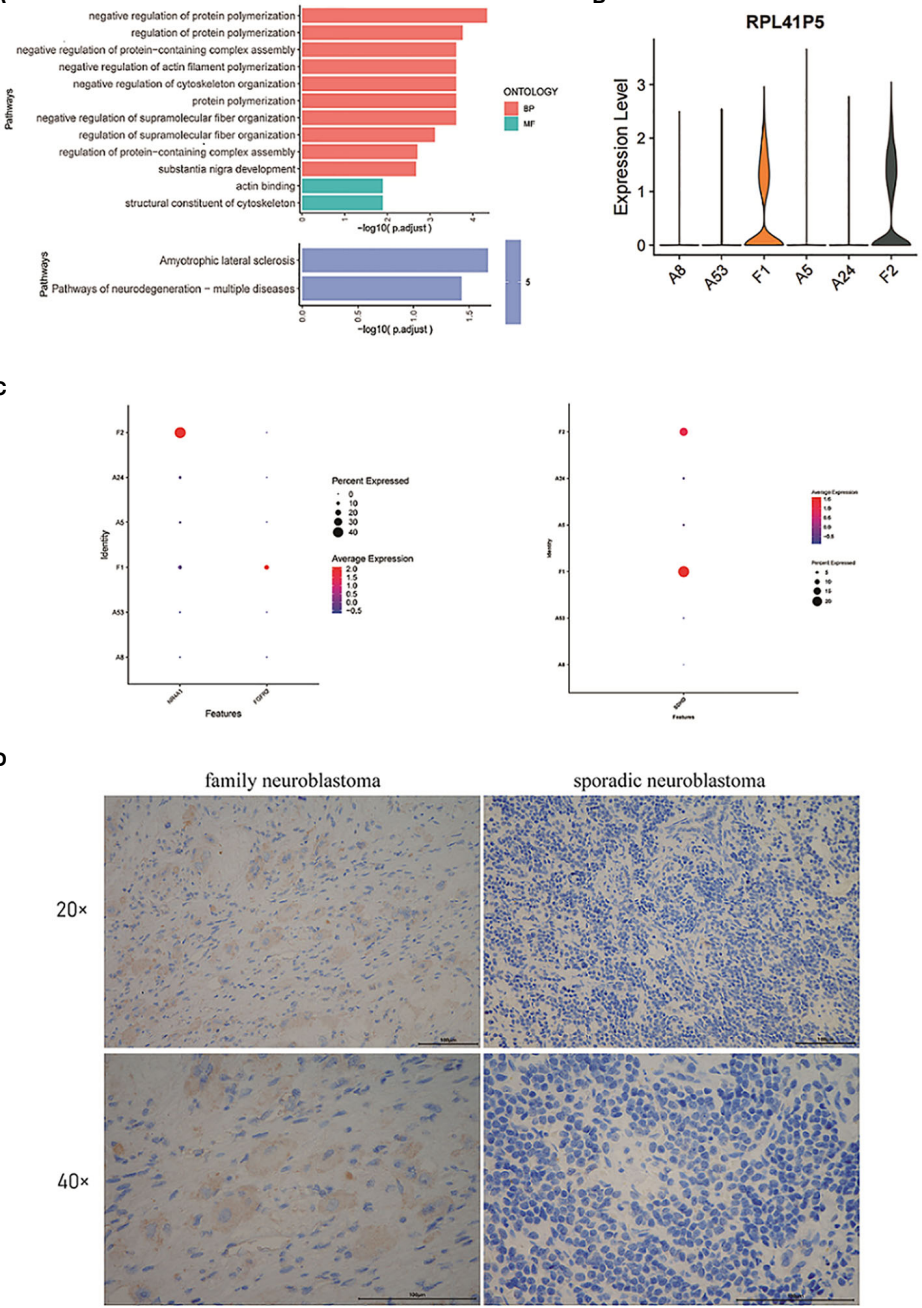
**(A)** GO and KEGG enrichment analysis of the genes in module 13. **(B)** The violin plot of the expression level of *RPL41P5*. **(C)** The dot plot of the expression level of *FGFR2*, *NR4A1* and *SDHD*. **(D)** The expression of *SDHD* at the protein level in tumor cells of family neuroblastoma and sporadic neuroblastoma as evaluated by immunohistochemistry.

Next, we performed Gene Ontology (GO) and Kyoto Encyclopedia of Genes and Genomes (KEGG) enrichment analysis of the module 13, which showed that the genes of module 13 were mainly enriched in the regulation of protein polymerization and amyotrophic lateral sclerosis (**Figure 3A**).

By differential expression gene analysis, we compared the DEGs between FNB and sporadic NB, and we focused on *SDHD*, *FGFR2*, and *NR4A1* (**Figure 3C**). Among them, *SDHD* may be associated with the development of family paraganglioma (42), which is also thought to originate from neural crest cells and has the same origin as NB. Therefore, the difference in *SDHD* protein expression between FNB and sporadic NB was further verified by immunohistochemistry (**Figure 3D**). In FNB, two cases (2/4, 50%) were positive for *SDHD* protein expression, whereas in sporadic neuroblastoma, all were negative for *SDHD* protein, and the immunohistochemical results further confirmed the difference in *SDHD* expression between FNB and sporadic neuroblastoma (**Supplementary Figure 2**). In addition, as genetic determinants of familial disease, *FGFR2* and *NR4A1* were associated with familial breast cancer and familial Crohn’s disease, respectively, and we similarly identified significant differences between groups, but *FGFR2* was expressed at lower levels in FNB and none in sporadic NB. Finally, we also identified the pseudogene *RPL41P5* (**Figure 3B**), but the pseudogene is currently considered non-functional.

### 3.4 Endothelial cells in familial neuroblastoma as a possible source of NEs

**Figure 4:**
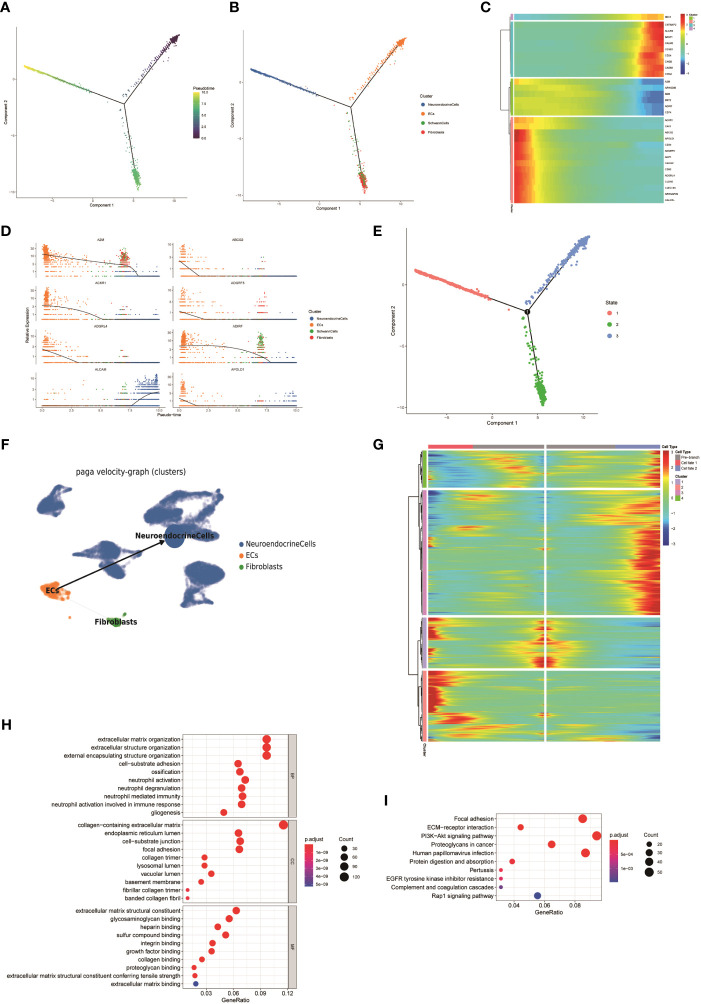
**(A, B)** The Monocle 2 trajectory plot shows the dynamics of ECs, fibroblasts, Schwann cells, and NEs. **(C)** Heatmap hierarchical clustering shows changes in the expression of genes in pseudo-time trajectories. **(D)** DEGs along the pseudo-time curve. **(E)** Distribution of cells in different states. **(F)** PAGA trajectory analysis of NEs, fibroblasts, and ECs(the thickness of the line indicates the strength of the differentiation relationship). **(G)** Changes in the expression level of DEGs during differentiation and clustering of similar genes. **(H, I)** GO and KEGG enrichment analyses of the cluster 2.

To understand the potential origin of NEs in FNB, we used pseudo-time trajectory analysis based on the Monocle 2 algorithm to distinguish whether there was a malignant transformation relationship between ECs, fibroblasts, Schwann cells, and NEs in FNB. The results showed that ECs have the potential to simultaneously evolve into NEs, fibroblasts, and Schwann cells (**Figures 4A, B**, **Supplementary Figure 3**). However, the number of Schwann cells was too small for subsequent analysis. Heatmap hierarchical clustering analysis demonstrated the changes of DEGs with the progression of the pseudo-time, and with the increase of the pseudo-time, *CHGB, CNTNAP2, ALCAM, BASP1, CALM2, CCND1, CD24, CADM1, CHGA*, and other genes increased in expression with increasing pseudo-time (**Figures 4C–E**). We next applied partition-based graph abstraction (PAGA) trajectory analysis to further clarify the differentiation direction of NEs, fibroblasts, and ECs, and the results (**Figure 4F**) were consistent with the previous trajectory analysis, in addition, the differentiation potential of endothelial cells to NEs was stronger than that of endothelial cells to fibroblasts, and finally, we used the branched expression analysis modeling (Beam) algorithm to investigate the gene expression changes during differentiation(**Figure 4G**). With the Beam algorithm, we clustered genes with similar expression patterns during differentiation into four clusters, from top to bottom, 4, 3, 1, and 2, respectively, from Pre-branch to cell fate1 representing the direction of differentiation of ECs to NEs, and from Pre-branch to cell fate2 representing the direction of differentiation of ECs to fibroblasts. We then performed GO and KEGG enrichment analyses on each cluster. We noticed that the DEGs expressed in the cluster 2 (ECs to NEs differentiation) were mainly enriched in collagen-containing extracellular matrix as well as PI3K-Akt signaling pathway (**Figures 4H, I**). The PI3K-AKT signaling pathway promotes the proliferation and growth of neuroblastoma (43).

### 3.5 FNB has a complex tumor microenvironment

**Figure 5:**
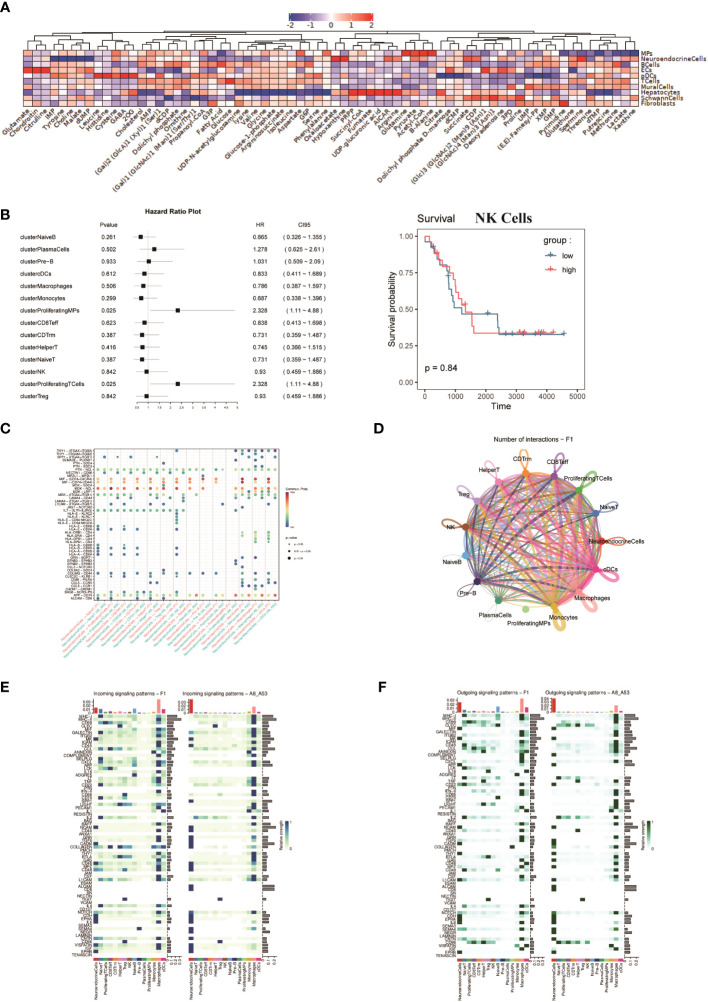
**(A)** Average metabolic heatmap of different cell types in different metabolites. **(B)** Survival analysis of the top100 genes for immune cell subpopulations, using NK cells as an example. **(C)** Point diagram of ligand-receptor analysis for different cell types. **(D)** The network of cell-to-cell ligand-receptor pairs by CellChat analysis. **(E, F)** Heatmap of signaling pathways respectively as ligand cells and receptor cells.

To assess the metabolic heterogeneity of the various cell types in the FNB, we performed single-cell flux estimation analysis (scFEA) (44) on all cell subpopulations of the six samples, which showed that compared to all other cell subpopulations, NEs were highly correlated with the metabolism of spermine, oxaloacetate, and hypoxanthine. The results showed that NEs were highly correlated with the metabolism of spermine, oxaloacetate, and hypoxanthine, while the metabolism of IMP, tyrosine, choline, malate, and dUMP was at a lower level compared to all other cell subpopulations (**Figure 5A**). In addition, we did not observe high levels of lactate metabolism at the level of overall NEs, and the level of lactate metabolism in NEs was lower than that in T cells. In addition, it has been previously described that FNB has a higher proportion of immune cells, which also predicts a more active TME. Next, survival analysis (**Figure 5B**) was performed on selected top100 genes for each subpopulation of non-malignant cells, showing that a variety of immune effector cells, including natural killer (NK) cells, were not associated with prognosis, focusing only on Proliferating-MPs and Proliferating-T Cells, which were associated with poor prognosis, unlike previously observed (45, 46). While further subdivision of immune cells showed that the TME of FNB was mainly composed of M2-like macrophages, which is consistent with the results observed for sporadic neuroblastoma, CellChat analysis then was used to identify the interaction between ligand-receptor pairs and cells, and due to the low number of non-NE cells within the P2 group, P1 was only analyzed. The results showed that in FNB, NE cells communicate most closely with Monocytes via *MIF*-(*CD74*+*CXCR4*) and *MIF*-(*CD74+CD44*) (**Figure 5C**), and several ligand receptors including *MDK*-*NCL* have important roles in intercellular interactions in TME, which are potential therapeutic targets for NB (**Figures 5D–F**).

### 3.6 Identification of a prognostic related CAF subtype in FNB: Fib-4

**Figure 6:**
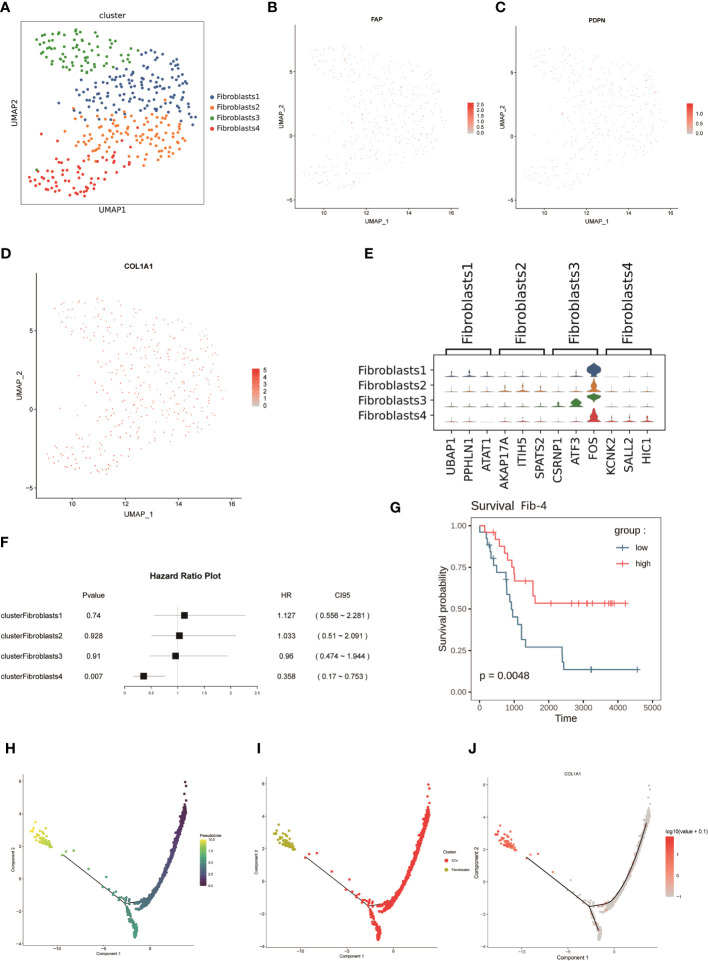
**(A)** The mechanical classification of fibroblasts resulted in a total of four subgroups. **(B-D)** UMAP plot for *FAP*,*PDPN*,and *COL1A1* in fibroblasts. **(E)** Violin plot of the expression of top genes in four clusters of fibroblasts. **(F, G)** Survival analysis of four subpopulations of fibroblasts. **(H, I)** Analysis of dynamic trajectory changes between endothelial cells and Fib-4 based on pseudo-time trajectories. **(J)** Changes in the expression level of *COL1A1* with the proposed-time.

Cancer-associated fibroblasts (CAF) are abundant in the tumor microenvironment and are associated with a variety of tumor biological behaviors including tumor invasion, drug resistance, and immune regulation (47, 48). A previous study using markers for CAF in breast cancer (48)defined CAF-S1 as well as CAF-S4 in human NB (49), and to further investigate FNB-CAF, we mechanistically classified fibroblasts in F1 (**Figure 1D**) and obtained a total of four subpopulations, namely Fib-1, Fib-2, Fib-3 and Fib- 4 (**Figure 6A**). In FNB, we did not identify the above two subpopulations (in which *αSMA*, *CD29*, and *PDGFRβ* were not expressed in the matrix, while *COL1A1* was expressed in all four subpopulations) (**Figures 6B-D**). The violin plot shows the highly expressed genes in each subpopulation (**Figure 6E**). Next, a survival analysis of the four subpopulations was performed, and the results showed that Fib-4 was associated with a good prognosis in NB (**Figures 6F, G**). Endothelial cells are also currently thought to be a source of CAF (50–52), so we further performed pseudo-time trajectory analysis of Fib-4 with endothelial cells in two FNB cases, and the Monocle 2 trajectory plot showed that in FNB, endothelial cells have the differentiation to Fib-4 potential (**Figures 6H-J**). In conclusion, a kind of prognostic related CAF subtype in FNB was identified by comprehensive bioinformatics, differentiated from endothelial cells, with good prognostic guidance in FNB.

## 4 Discussion

To investigate the genetic properties of FNBs, we performed scRNA-Seq on 35369 cells from six tumors including two FNB. Here, a detailed transcriptional atlas of FNB was created, demonstrating the unique network of cellular and molecular interactions of FNB. Metabolomic analysis revealed that NEs are highly correlated with the metabolism of spermine, oxaloacetate and hypoxanthine. Previous studies from our group (16) identified the potential for transformation between NEs and fibroblasts, while the latest results show that endothelial cells in FNB have the potential to differentiate into NEs and fibroblasts. Based on this, a prognostic related CAF phenotype was identified in FNB: Fib-4, a subpopulation of cells that may differentiate from endothelial cells, which is associated with a good prognosis and may be a potential therapeutic target.

By comparing gene expression levels between samples, we identified DEGs that may be associated with FNB. First of all, it is remarkable that we focused on a pseudogene: *RPL41P5*. Pseudogenes are known to be non-functional genomes, which cannot translate proteins (53, 54). Pseudogene-derived long noncoding RNA(lncRNA) is currently thought to have an important role in the development of human cancers (55), and a previous experiment in nude mice, as well as NB cell lines, confirmed that the pseudogene *DUXAP8* is associated with the progression and poor prognosis of NB (56). *SDHD*, identified by differential expression between groups, is currently thought to be associated with familial paraganglioma (57) as well as pheochromocytoma (58, 59). And both diseases are similar in origin to neuroblastoma, deriving from neural crest cells, so we speculate that *SDHD* may be associated with the development of familial neuroblastoma. Immunohistochemical analysis confirmed the expression of *SDHD* in FNB. Therefore, we suggest that *SDHD* may be related to FNB, which still needs further confirmation.

By Hotspot analysis and Jaccard similarity analysis of NEs, we identified a total of 15 modules, of which the module 13 from FNB was associated with better survival. Unfortunately, we did not identify identically expressed modules in the two FNB, whereas similarly expressed modules were found between sporadic neuroblastoma of similar tumor stages. The author believes that this phenomenon may be related to the small sample size of the FNBs analyzed, and secondly, if multiple samples and analyses of the same family line can be performed, there may be more desirable results.

By scFEA of NEs in all six samples, we found that NEs are associated with the metabolism of spermine, oxaloacetate, and hypoxanthine. F14512, a topoisomerase II inhibitor containing the spermine fraction, was found to have significant and long-lasting antitumor effects on NB and to have synergistic effects with cisplatin and carboplatin (60). The presence of pyruvate carboxylase (PC), which converts pyruvate to oxaloacetate, has now been demonstrated in NB (61). In addition, it is believed that cancer cells can use Lactate dehydrogenase(LDH) to reduce pyruvate to lactate in order to bypass oxidative phosphorylation (62), and the increased lactate level can enhance tumor angiogenesis and facilitate tumor growth (63, 64), but we found that the level of lactate metabolism in NEs is lower than that in T cells. We have for the first time mapped the metabolism of NB at the single cell level,and the mechanisms related to these metabolites will provide new instruments for the treatment of neuroblastoma.

Finally, we focused on the TME, and it is noteworthy that the TME of FNB is composed mainly of M2-like macrophages, and intercellular communication analysis likewise showed that macrophages are the most active in FNB. Finally, since only one of the six FNBs identified a certain number of fibroblasts, CAF was later identified in this FNB by a marker that has been reported in the literature, and four subpopulations were identified by mechanical classification, and survival analysis showed that Fib-4 was associated with a good survival prognosis. However, we did not identify CAF-S1 and CAF-S4 in the FNB as previously reported in the paper (49).

In summary, we depicted the transcriptional profile of FNB by single-cell RNA sequencing and identified genes that may be associated with FNB by differential expression analysis: *SDHD*.In addition, we identified a prognostic related CAF: Fib-4 group in FNB which was associated with good prognosis. Spermine, oxaloacetate, and hypoxanthine metabolites were found to be highly expressed in NEs.The mechanisms depicted for the differentiation direction of each cell subpopulation and intergroup communication may provide new ideas for the treatment of neuroblastoma.

## 5 Materials and methods

### 5.1 Patients and tumor tissues

Tumor tissues were obtained from children who were diagnosed with NB in the department of pediatric surgical oncology of children’s hospital of Chongqing medical university. Approval was obtained from the institutional ethics committee at our hospital. The fresh tumor tissues were rinsed with normal saline after surgical resection to remove blood cells. Then, the non-necrotic parts of tumor tissues (0.3 - 0.5 m^3^ per sample) were moved out, stored in 1 ml GEXSCOPE^®^ Tissue Preservation Solution (Singleron, China) and transported to the Singleron lab immediately.

### 5.2 Tissue dissociation and preparation

The tumor tissues were washed with Hanks balanced salt solution (HBSS) for 3 times and cut into small pieces (1~2 mm), and put into 2 ml GEXSCOPE ^®^ In tissue dissociation solution (Singleron), stirred gently and continuously at 37 °C for 15 min. After digested, the samples were iltered with 40-micron sterile strainers and centrifuged at 800×g for 5 min. Then, resuspended the samples in 1 ml phosphate buffer (PBS) (HyClone) which added 2 ml GEXSCOPE^®^ red blood cell lysis buffer (Singleron) at 25 °C for 5-8 min to remove red blood cells. The above mixture was centrifuged at 500 × g for 5 minutes to precipitate cells. Then resuspended cells with PBS. Finally, stained the samples with Trypan Blue to evaluate the cell viability microscopically.

### 5.3 Single-cell RNA sequencing

The concentration of single cell suspensions was adjusted to 1 x 10^5^ cells/mL. Then, the suspensions were loaded onto microfluidic chip, and scRNA-seq libraries were constructed according to the instructions of Singleron (GEXSCOPE^®^ Single-Cell RNA Library Kit, Singleron Biotechnologies). Individual libraries were diluted to 4 nM and pooled to sequence on an Illumina HiSeq X with 150-bp paired-end reads.

### 5.4 Primary analysis of raw read data

Raw reads from scRNA-seq were processed to generate gene expression matrixes using CeleScope (https://github.com/singleron-RD/CeleScope) v1.9.0 pipeline. Briefly, raw reads were first processed with CeleScope to remove low quality reads with Cutadapt v1.17 to trim poly-A tail and adapter sequences. Cell barcode and UMI were extracted. After that, we used STAR v2.6.1a (65) to map reads to the reference genome GRCh38 (ensembl version 92 annotation). UMI counts and gene counts of each cell were acquired with featureCounts v2.0.1 (66) software, and used to generate expression matrix files for subsequent analysis.

### 5.5 Quality control, dimension-reduction and clustering

Scanpy v1.8.1 was used for quality control, dimensionality reduction and clustering under Python 3.7. For each sample dataset, we filtered expression matrix by the following criteria: 1) cells with gene count less than 200 or with top 2% gene count were excluded; 2) cells with top 2% UMI count were excluded; 3) cells with mitochondrial content 20% were excluded; 4) genes expressed in less than 5 cells were excluded. The raw count matrix was normalized by total counts per cell and logarithmically transformed into normalized data matrix. Top 2000 variable genes were selected by setting flavor = ‘seurat’. Principle Component Analysis (PCA) was performed on the scaled variable gene matrix, and top 20 principle components were used for clustering and dimensional reduction Batch effect between samples was removed by Harmony (67). Finally, UMAP algorithm was applied to visualize cells in a two-dimensional space.

Scanpy v1.8.1 was used for further clustering analysis of Fibroblast. Top 2000 variable genes were selected for PCA analysis. The first 6 principle components and resolution parameter 0.8 were used with louvain algorithm to generate 4 cell clusters.

Differentially expressed genes (DEGs) and pathway enrichment analysis, and cell type annotation

DEGs were determined as genes expressed in more than 10% of the cells in a cluster with an average log (Fold Change) of greater than 0.25, and were selected by scanpy rank_genes_groups function based on the Wilcox likelihood-ratio test with default parameters. The Gene Ontology (GO) and Kyoto Encyclopedia of Genes and Genomes (KEGG) analysis were used to investigate the potential functions of DEGs with the “clusterProfiler” R package version 3.16.1. *P*-value < 0.05 was considered statistically significant. The cell type identity of each cluster was determined with the expression of canonical markers found in the DEGs using SynEcoSys database. Violin plots displaying the expression of markers used to identify each cell type were generated by Seurat v3.1.2 Vlnplot.

### 5.6 Trajectory analysis

Cell differentiation trajectory was reconstructed with Monocle2 (68). Next, highly-variable genes (HVGs) were used to sort cells in order of spatial‐temporal differentiation. DDRTree was used to perform dimension-reduction. Finally, the trajectory was visualized by plot_cell_trajectory function. To run PAGA (69), a symmetrized kNN-like graph based on PCA data was construct by scanpy.tl.paga, using the approximate nearest neighbor search within UMAP. For each partitioning, a PAGA graph was generated using the connectivity.

### 5.7 scRNA-seq based CNA detection

The InferCNV package (70) were used to evaluate the CNVs in NE cells Schwann cells, endothelial cells, and fibroblasts. T cells, B cells and myeloid cells were regarded as baselines to estimate the CNAs of malignant cells. Genes expressed in more than 20 cells were classified based on their loci on each chromosome. The relative expression values were centered to 1, using 1.5 standard deviations from the residual-normalized expression values as the ceiling. A slide window size of 101 genes was used to normalize the relative expression on each chromosome in order to remove the effect of gene-specific expression.

Each p- or q-arm level change can be simply converted to an equivalent CNV according to its location by considering genomic cytoband information. Each CNV was annotated as a gain or loss. Then, subclones containing the same arm-level CNVs were folded, and trees were reconstructed to represent the architecture of subclonal CNV.

### 5.8 Functional gene module analysis

Hotspot (37) was used to identify functional gene modules which illustrate heterogeneity within NEs subpopulations. Briefly, we used the ‘danb’ model and selected the top 500 genes with highest autocorrelation zscore for module identification. Modules were then identified using the create_modules function, with min_gene_threshold =15 and fdr_threshold = 0.05. Module scores were calculated by using calculate_module_scores function. The Jaccard similarity coefficient was calculated for comparing transcriptional similarity between cell types with hotspot modules genes (15).

### 5.9 Cell-cell interaction analysis (CellChat)

CellChat (version 0.0.2) (71) was used to analyze the intercellular communication networks from scRNA-seq data. A CellChat object was created using the R package process. Cell information was added into the meta slot of the object. The ligand-receptor interaction database was set, and the matching receptor inference calculation was performed.

### 5.10 Immunohistochemistry (IHC)

IHC was used to analyze the protien expression of SDHD in tumor tissue. Paraffin sections of 4 familial NB and 4 sporadic NB were collected. The paraffin sections were processed with baking, dewaxing, sodium citrate antigen repair, 3% H_2_O_2_ incubation and 0.5% BSA closure. Then sections were incubated with anti-SDHD (Abcam, ab189945), and corresponding secondary antibodies (ZSGB-Bio, PV-9001). DBA color development was carried out according to the instructions of the immunohistochemistry kit (ZSGB-Bio, ZLI 9019). Finally, the sections were stained with hematoxylin, sealed with neutral gum, and observed under a light microscope.

### 5.11 Metabolic fluxomes and abundances analysis:scFEA

scFEA (v1.1.2) (45) is a computational method for inferring cellular metabolic fluxomes and metabolite abundances from scRNA-seq data using flux balance constraints based on novel probabilistic models. All cell types were calcuated the metabolic Fluxomes and Abundances by scFEA in python. After calculating metabolic flux and metabolite abundances, the 70 metabolic modules and all metabolites were selected for visualization by heatmap.

## Data availability statement

The raw data supporting the conclusions of this article will be made available by the authors, without undue reservation.

## Ethics statement

The studies involving humans were approved by the Institutional Review Board of the Children’s Hospital of Chongqing Medical University. The studies were conducted in accordance with the local legislation and institutional requirements. Written informed consent for participation in this study was provided by the participants’ legal guardians/next of kin.

## Author contributions

SW was responsible for the general conception of the study and for revising the manuscript. YZ was responsible for data collection and data analysis and wrote the manuscript. YM was responsible for data analysis as well as experiments. QL was responsible for part of the data analysis. YD, LP, and JZ were involved in the sample collection. ZZ and CL were responsible for the guidance and participated in the sample collection. All authors contributed to the article and approved the submitted version.

## Supplementary material

Supplementary Figure 1 The expression of Ki-67 **(A, C)** and S-100 **(B, D)** in tumor cells of F2 and F2’s sister (**A, B** belong to F2; **C, D** belong to F2’s sister) by immunohistochemistry

Supplementary Figure 2 The expression of SDHD at the protein level in tumor cells of 4 family neuroblastomas **(A/a, F/f, G/g, H/h)** and 4 sporadic neuroblastomas **(B/b, C/c, D/d, E/e)** by immunohistochemistry.

Supplementary Figure 3 The Monocle 2 trajectory plot shows the dynamics of six samples **(A)** and four cell types **(B)**.
