# Supplementary figures and images for: Single-cell transcriptome sequencing reveals tumor heterogeneity in family neuroblastoma
Source: Front Immunol. 2023 Sep 18;14:1197773. doi: 10.3389/fimmu.2023.1197773 (PMC10543897; doi:10.3389/fimmu.2023.1197773)

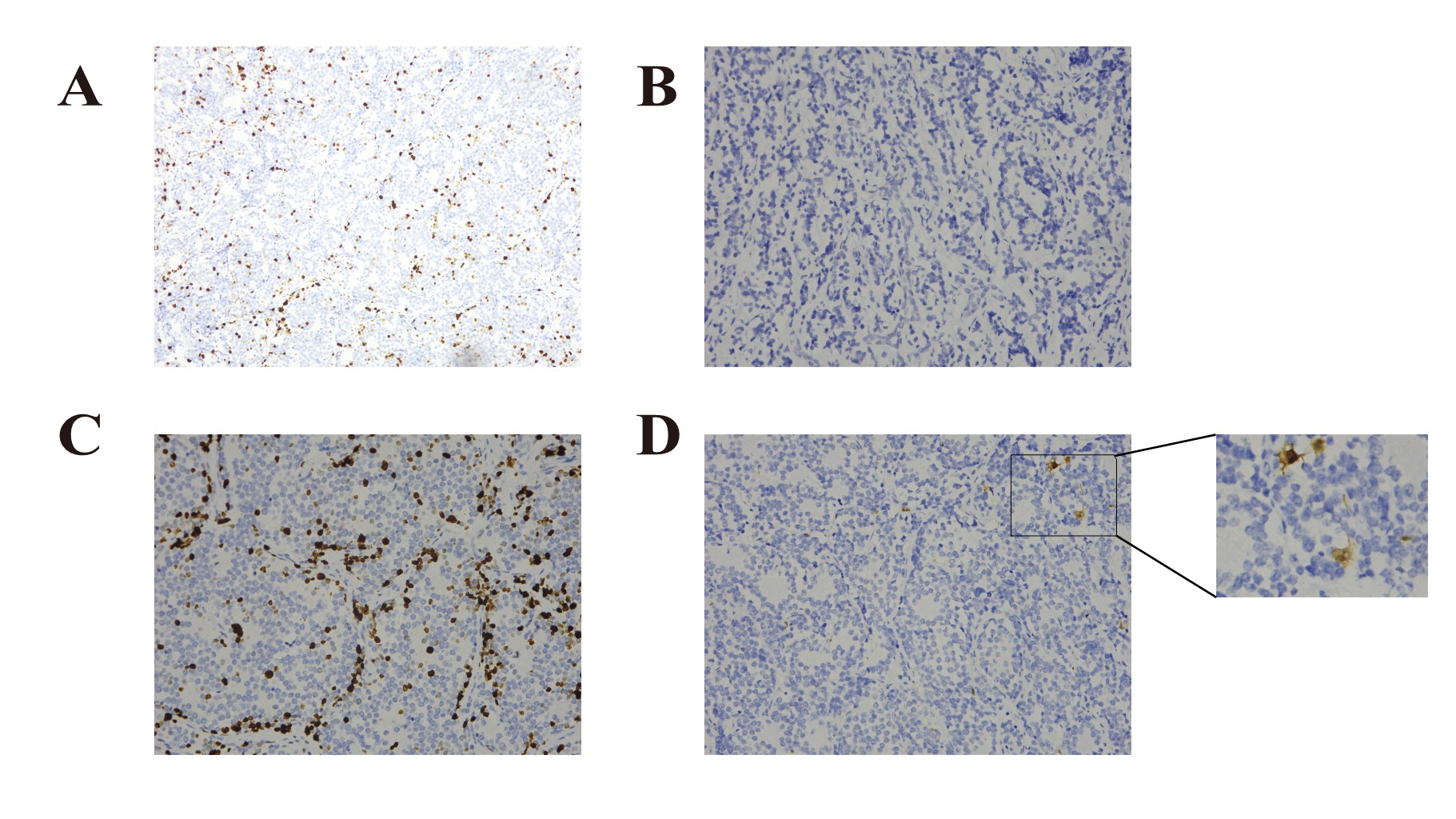

Supplement: Supplementary Figure 1 — The expression of Ki-67 (A, C) and S-100 (B, D) in tumor cells of F2 and F2’s sister (A, B belong to F2; C, D belong to F2’s sister) by immunohistochemistry [file Image_1.jpeg]

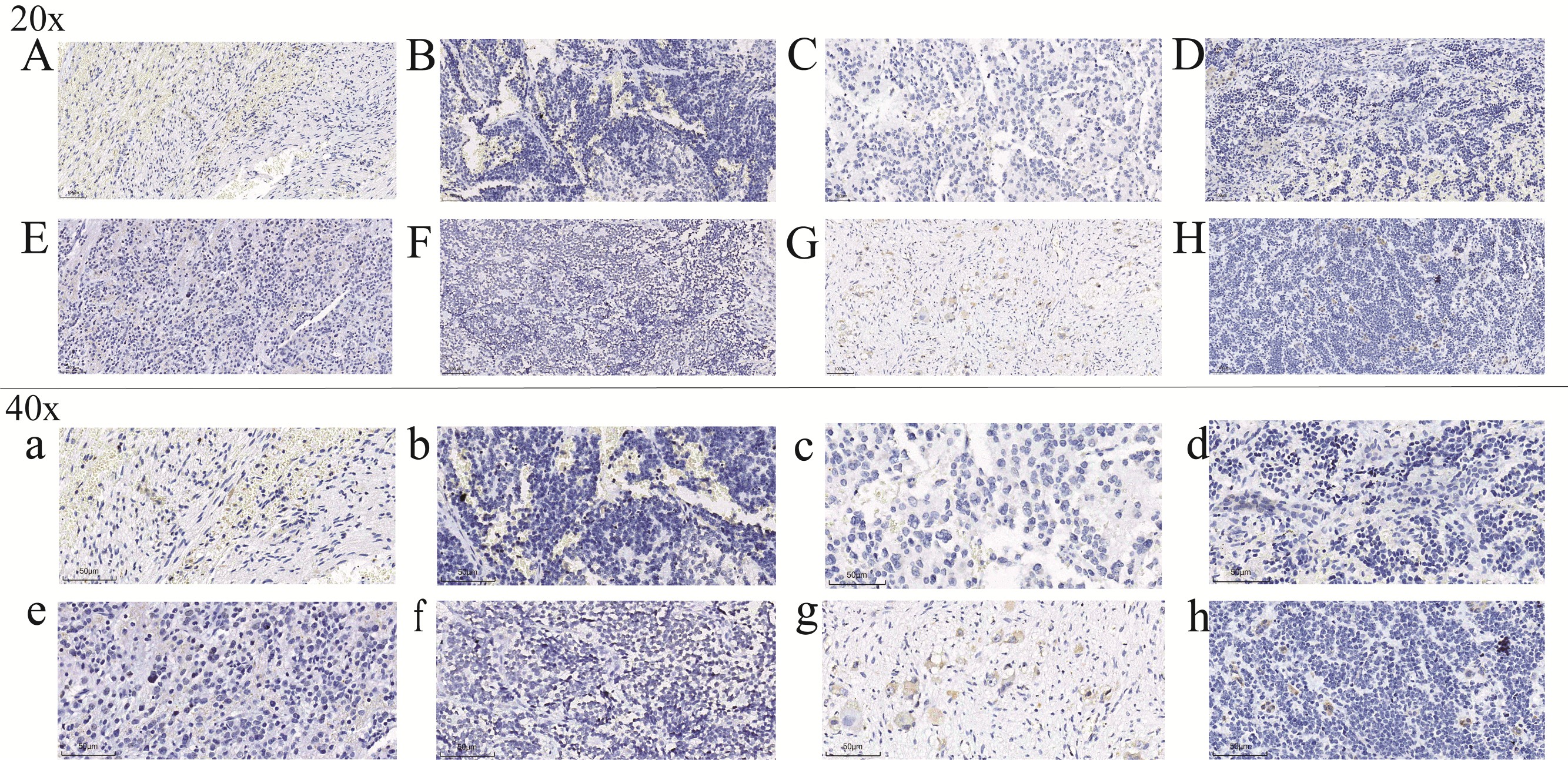

Supplement: Supplementary Figure 2 — The expression of SDHD at the protein level in tumor cells of 4 family neuroblastomas (A/a, F/f, G/g, H/h) and 4 sporadic neuroblastomas (B/b, C/c, D/d, E/e) by immunohistochemistry. [file Image_2.jpeg]

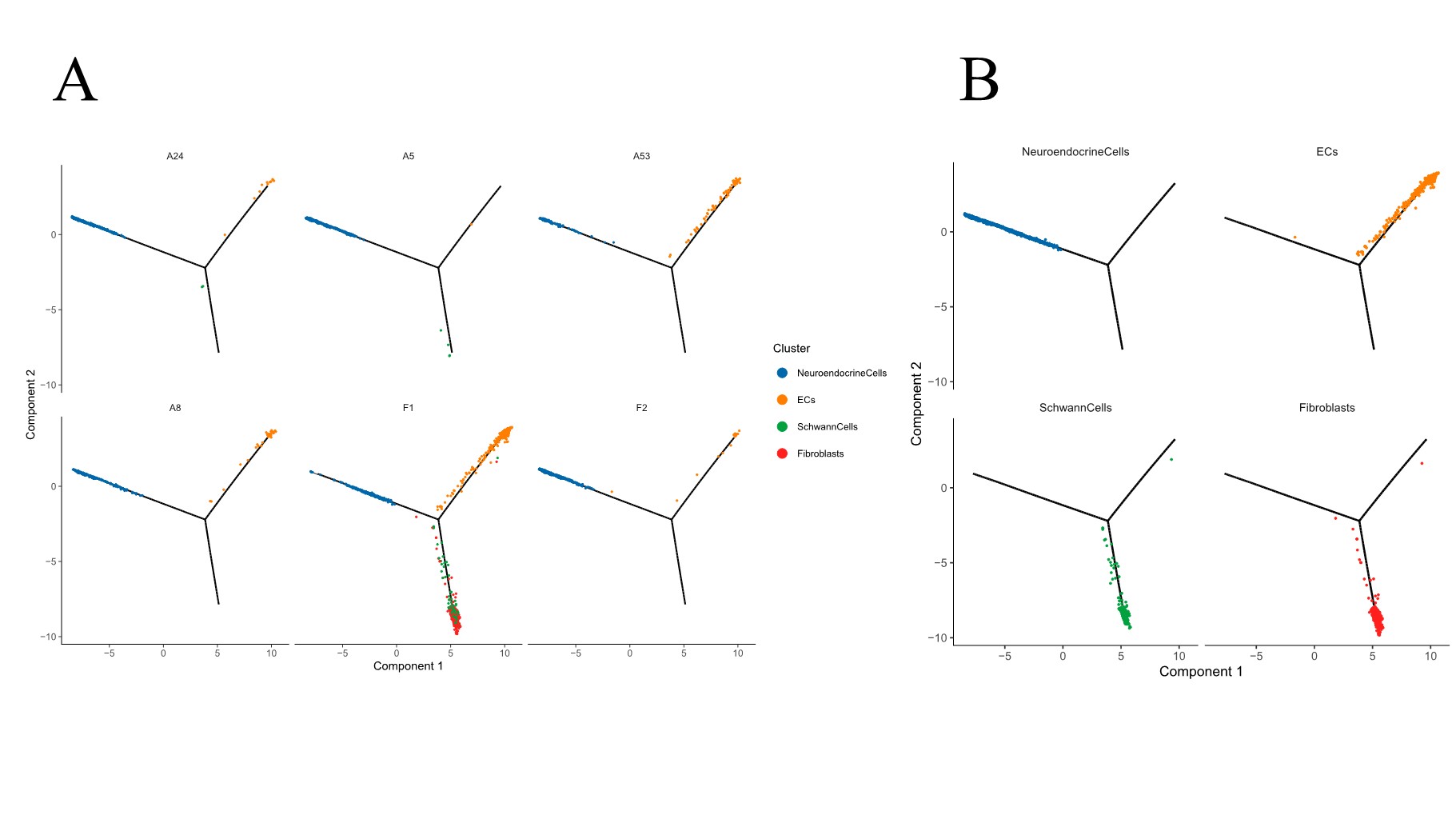

Supplement: Supplementary Figure 3 — The Monocle 2 trajectory plot shows the dynamics of six samples (A) and four cell types (B). [file Image_3.jpeg]

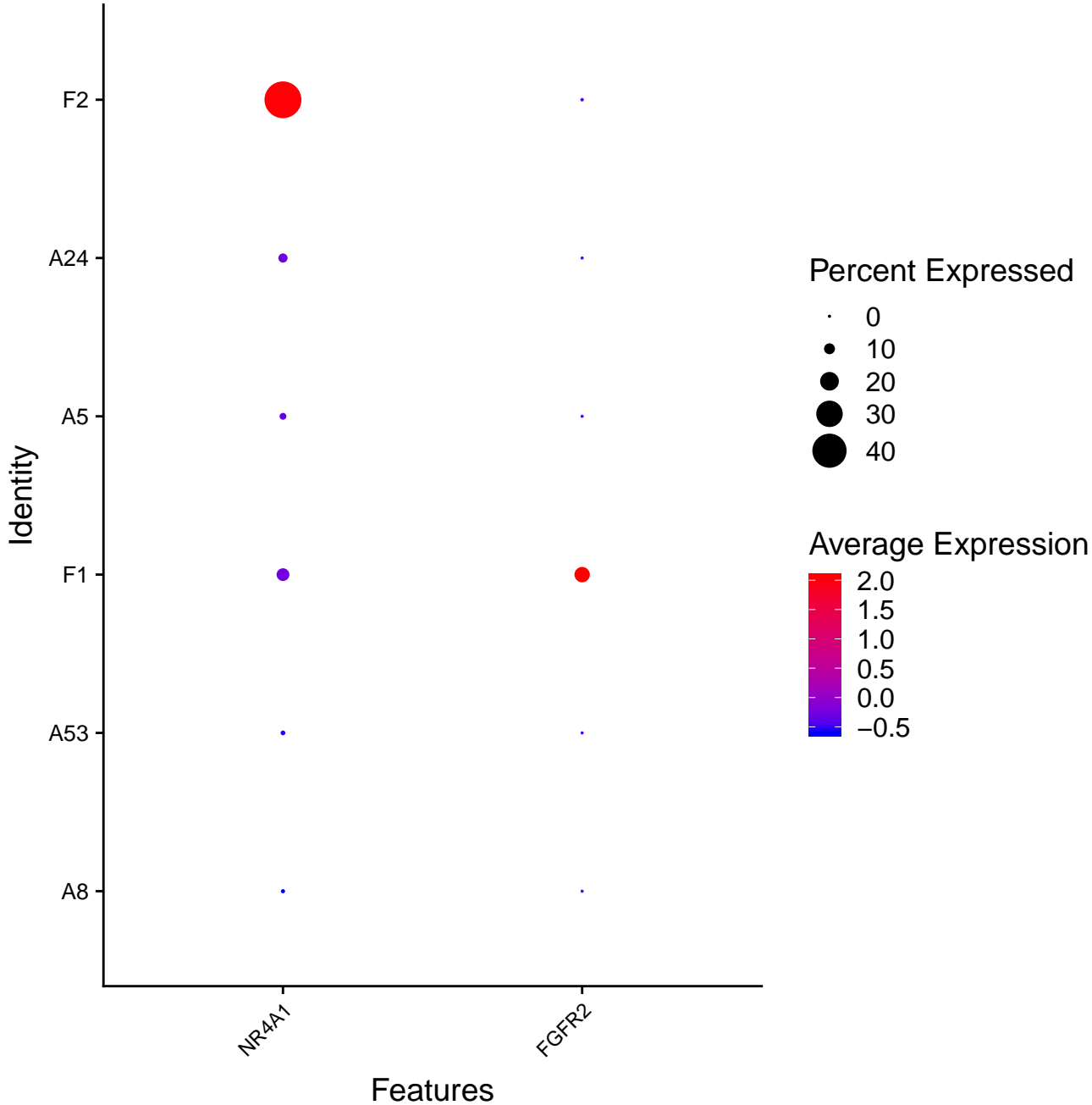

Supplement: Supplementary file 6 [file DataSheet_3.pdf]

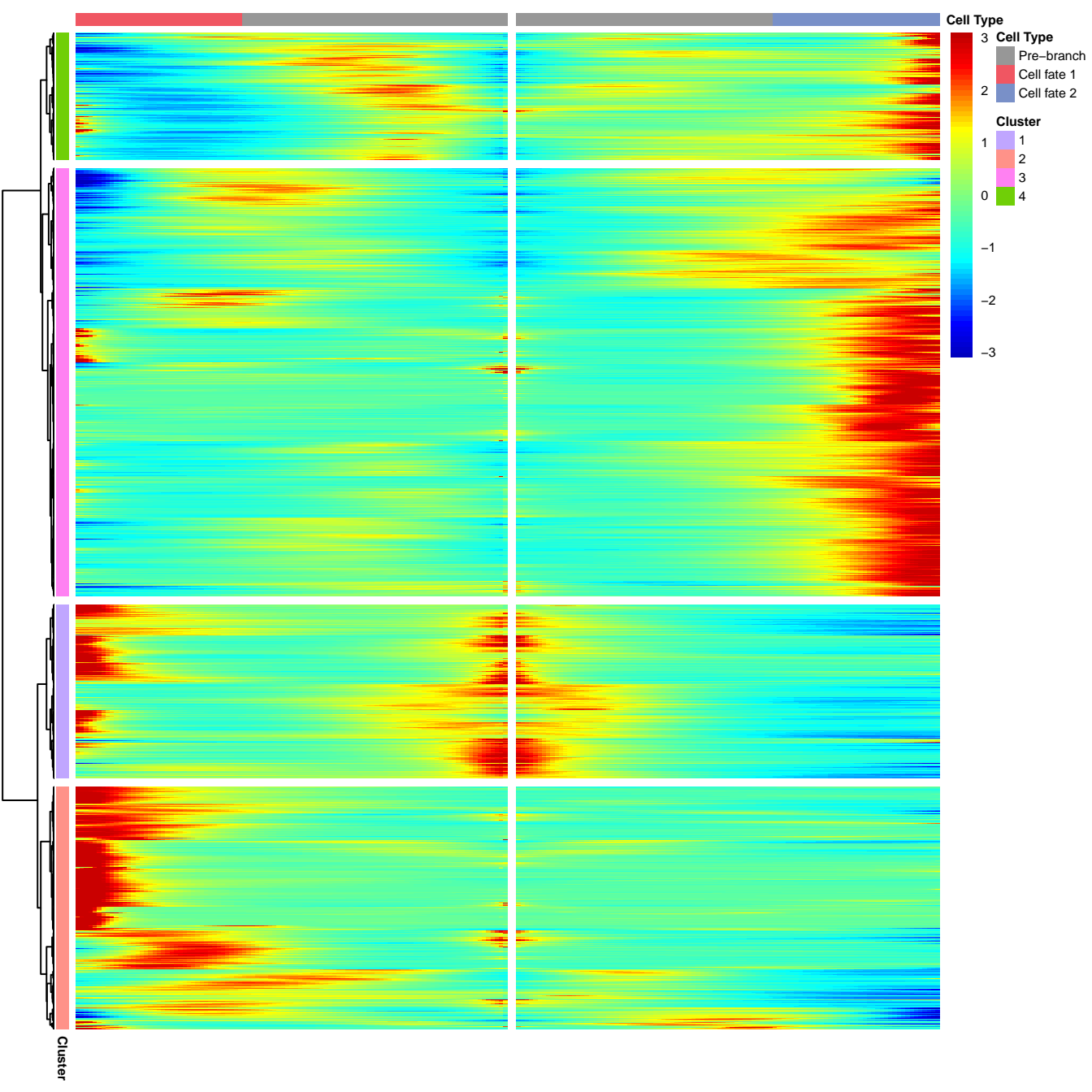

Supplement: Supplementary file 7 [file DataSheet_4.pdf]

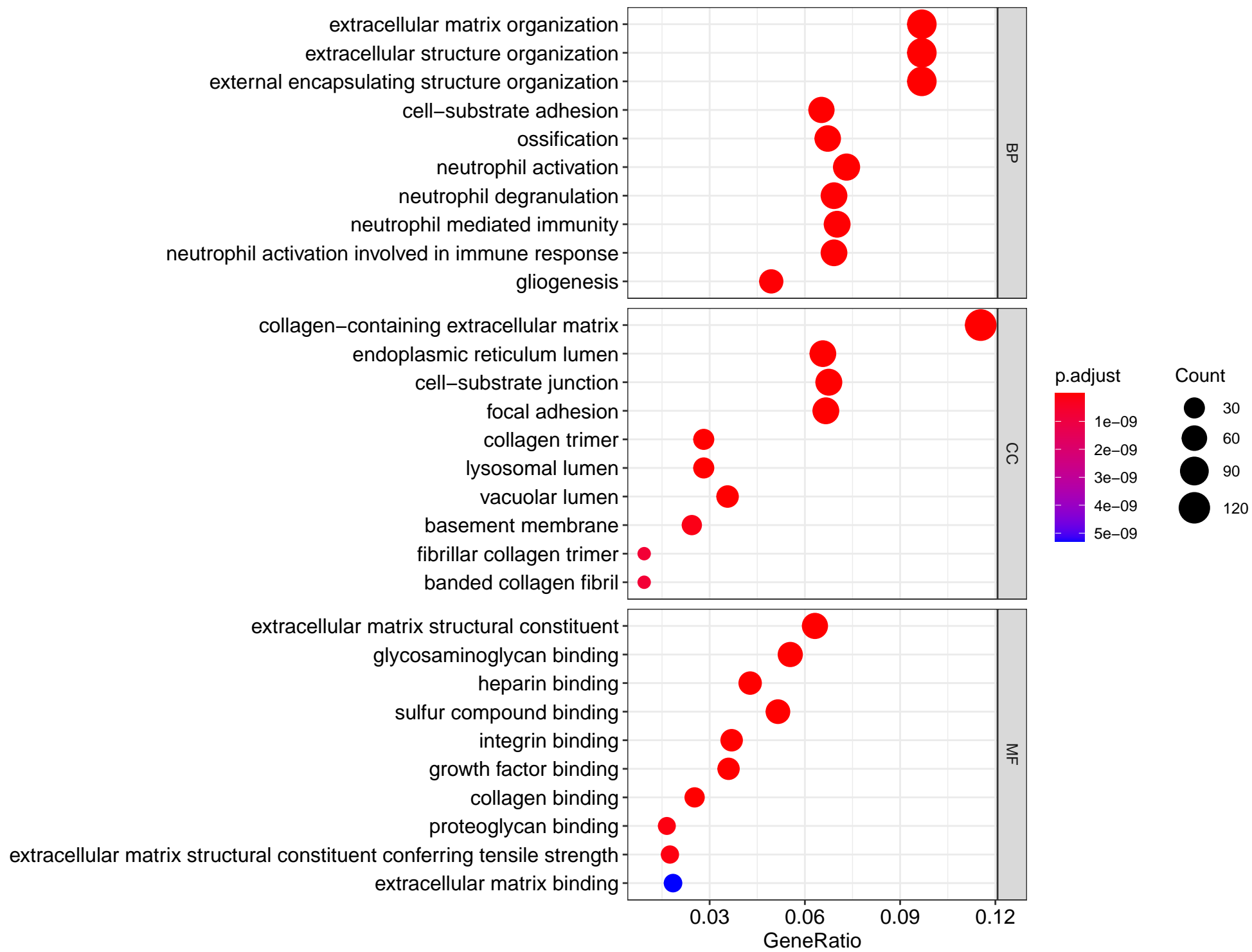

Supplement: Supplementary file 8 [file DataSheet_5.pdf]
