# Supplementary material for: Single-cell transcriptome sequencing reveals tumor heterogeneity in family neuroblastoma
Source: Front Immunol. 2023 Sep 18;14:1197773. doi: 10.3389/fimmu.2023.1197773 (PMC10543897; doi:10.3389/fimmu.2023.1197773)
Supplement: Supplementary file 4 [file DataSheet_1.pdf]

```
##script_for_python_based_analysis
import argparse
import numpy as np
import pandas as pd
import scanpy as sc
import harmonypy as hm
from matplotlib import pyplot as plt
import matplotlib
matplotlib.use("Agg")

##############################################################################################
###################
# multi-sample merge and analysis by scanpy
infor = 'samplt_table.txt'    # sample information
intab = pd.read_csv(infor, sep="\t")
maxgenes = 0.98
maxumis = 0.98
mtfilter = 20
rmbatch = 'F'
resolution = 1.2
clustcol = ['#0067AA','#FF7F00','#00A23F','#FF1F1D','#A763AC','#B45B5D','#FF8AB6','#B6B800',

'#01C1CC','#85D5F8','#FFC981','#C8571B','#727272','#EFC800','#8A5626','#502E91',

'#59A4CE','#344B2B','#FBE29D','#FDD6E6','#849C8C','#F07C6F','#000101','#FF4500',

'#6959CD','#FF8C00','#ADFF2F','#A020F0','#2F4F4F','#FFD700','#EE1289','#8B2500',
            '#4682B4','#FFDAB9','#708090','#836FFF']

# merge samples matrix
adatas = []
for index, line in intab.iterrows():
    adata = sc.read_10x_mtx(line.path, var_names="gene_symbols", cache=False,
make_unique=True)
    adata.var_names_make_unique()
    adata.obs["sample"] = line.spname
    add_names = line.index.drop(["spname", "path", "datatype"])
    for tag in add_names: adata.obs[tag] = line[tag]
    barcode = []
    for bar in adata.obs.index:
        newbarcode = line.spname + "_" + bar
        barcode.append(newbarcode)
    adata.obs.index = barcode
```

```
    sc.pp.filter_cells(adata, min_genes=200)
    sc.pp.filter_cells(adata, min_counts=200)
    maxgenes_new = maxgenes if maxgenes > 1 else np.percentile(adata.obs["n_genes"], 100 * maxgenes) + 1
    maxumis_new = maxumis if maxumis > 1 else np.percentile(adata.obs["n_counts"], 100 * maxumis) + 1
    sc.pp.filter_cells(adata, max_genes=maxgenes_new)
    sc.pp.filter_cells(adata, max_counts=maxumis_new)
    sc.pp.filter_genes(adata, min_cells=5)
    adatas.append(adata)

adata = sc.concat(adatas, join="outer")
adata.var_names_make_unique()
adata.X = np.nan_to_num(adata.X)

# filter high mt cells
adata.var["mt"] = adata.var_names.str.contains("^[Mm][Tt]-")
sc.pp.calculate_qc_metrics(adata, qc_vars=["mt"], percent_top=None, log1p=False, inplace=True)
adata = adata[adata.obs.pct_counts_mt < mtfilter, :]
adata.obs.rename(columns={"n_genes": "nFeature_RNA", "n_counts": "nCount_RNA", "pct_counts_mt": "percent_mt"}, inplace=True)
adata.raw = adata
adata.layers["counts"] = adata.X.copy()

#
sc.pp.normalize_total(adata, target_sum=1e4)
sc.pp.log1p(adata)
adata.layers["data"] = adata.X.copy()
sc.pp.highly_variable_genes(adata, n_top_genes=2000, flavor="seurat")
sc.pl.highly_variable_genes(adata)
plt.savefig(f'HVG.png')
plt.close()

sc.pp.scale(adata, max_value=10)
sc.tl.pca(adata, svd_solver="arpack", use_highly_variable=True)
sc.pl.pca_variance_ratio(adata, log=False, n_pcs=50)
plt.savefig(f'PCElbowPlot.png')
plt.close()

# remove batch by harmony
if rmbatch == "T":
    ho = hm.run_harmony(adata.obsm["X_pca"], adata.obs, ['sample'])
    res = pd.DataFrame(ho.Z_corr.T)
    res.columns = ["harmony_{}".format(i + 1) for i in range(res.shape[1])]
```

```
        res.index = adata.obs.index
        adata.obsm["X_harmony"] = np.array(res)
        adata.obsm["X_pca"] = np.array(res)

sc.pp.neighbors(adata, n_neighbors=15, n_pcs=30)
sc.tl.louvain(adata, resolution=resolution)
sc.tl.leiden(adata, resolution=resolution)
adata.obs["cluster"] = adata.obs['louvain']
max_num = max(pd.to_numeric(adata.obs["cluster"]))
adata.rename_categories("cluster", list(map(str, list(range(1, max_num + 2)))))
sc.tl.umap(adata)
sc.tl.tsne(adata)
adata.X = adata.layers["data"]
adata.write(f"cluster_result.h5ad")

# anno celltype
adata = sc.read_h5ad(f"cluster_result.h5ad")
used_cell = adata.obs
ctfile = pd.read_csv('cluster_celltype.txt', sep="\t", dtype='str')
ctfile.index = ctfile.cluster
ctfile = ctfile.loc[ctfile.index.isin(adata.obs.cluster.unique()), ]
used_cell = used_cell[used_cell['cluster'].isin(ctfile.index)]
adata = adata[used_cell.index, ]
adata.obs['cluster'] = adata.obs['cluster'].astype(str)
adata.obs['cluster'] = pd.array(ctfile.loc[adata.obs.cluster, 'celltype'])
adata.obs['cluster'] = adata.obs['cluster'].astype('category')
adata.obs['cluster'].cat.set_categories(ctfile.celltype.unique(), inplace=True)
adata.write(f"celltype_result.h5ad")

# reduct cluster on plot
sc.pl.umap(adata, color="cluster", legend_loc="on data", palette=clustcol, legend_fontoutline=1)
plt.gca().set_box_aspect(1)
plt.savefig(f"labumap.png", bbox_inches="tight", dpi=300)
plt.savefig(f"labumap.pdf", bbox_inches="tight", dpi=300)
plt.close()
# reduct cluster with label right
sc.pl.umap(adata, color="cluster", legend_loc="right margin", palette=clustcol)
plt.gca().set_box_aspect(1)
plt.savefig(f"rlabumap.pdf", bbox_inches="tight")
plt.savefig(f"rlabumap.png", bbox_inches="tight", dpi=300)
plt.close()
# reduct sample
sc.pl.umap(adata, color="sample", legend_loc="right margin", palette=clustcol)
plt.gca().set_box_aspect(1)
```

```
plt.savefig(f"umap_samples.pdf", bbox_inches="tight")
plt.savefig(f"umap_samples.png", bbox_inches="tight", dpi=300)
plt.close()

# get diff genes
sc.tl.rank_genes_groups(adata, "cluster", method="wilcoxon", pts=True, use_raw=False)
groups = adata.uns["rank_genes_groups"]["names"].dtype.names
for i in groups:
	de_i = sc.get.rank_genes_groups_df(adata, group=i)
	de_i = de_i[de_i.logfoldchanges.abs() >= 1]
	de_i = de_i[(de_i["pct_nz_group"] >= 0.1) | (de_i["pct_nz_reference"] >= 0.1)]
	de_i.to_csv(f"cluster{i}_diffgenes.xls", index=False, sep="\t")

# plot genes
features = []
sc.pl.heatmap(adata, features, groupby="cluster", swap_axes=True, show_gene_labels=True, use_raw=False, standard_scale="var")
plt.savefig(f"TopMarkergeneHeatmap.pdf", bbox_inches="tight")
plt.savefig(f"TopMarkergeneHeatmap.png", bbox_inches="tight", dpi=300)

sc.pl.dotplot(adata, features, 'cluster', use_raw=False, swap_axes=False)
plt.savefig(f"TopMarkergenedotplot.pdf", bbox_inches="tight")
plt.savefig(f"TopMarkergenedotplot.png", bbox_inches="tight", dpi=300)
plt.close()

sc.pl.tracksplot(adata, features, groupby='cluster', use_raw=True)
plt.savefig(f"TopMarkergeneTracksplot.pdf", bbox_inches="tight")
plt.savefig(f"TopMarkergeneTracksplot.png", bbox_inches="tight", dpi=300)
plt.close()

sc.pl.stacked_violin(adata, features, groupby="cluster", use_raw=False, row_palette=clustcol)
plt.savefig(f"TopStackedViolin.pdf", bbox_inches="tight")
plt.savefig(f"TopStackedViolin.png", bbox_inches="tight", dpi=300)
plt.close()


############################################################################################
##########################
# hotspot script for gene module analysis
import hotspot

hs_obj = hotspot.Hotspot(adata, layer_key='counts', model='danb',
                         latent_obsm_key='X_pca', umi_counts_obs_key='nCount_RNA')
hs_obj.create_knn_graph(weighted_graph=False, n_neighbors=30)
```

```
hs_results = hs_obj.compute_autocorrelations()
hs_results.head(15)
hs_genes = hs_results.loc[hs_results.FDR < 0.05].sort_values('Z', ascending=False).head(int(500)).index
local_correlations = hs_obj.compute_local_correlations(hs_genes)
modules = hs_obj.create_modules(min_gene_threshold=15, core_only=True, fdr_threshold=0.05)
hs_obj.plot_local_correlations(vmin=-12, vmax=12, mod_cmap='tab20')
pdf = 'Correlation_heatmap.pdf'
plt.savefig(pdf, bbox_inches="tight")
results = hs_obj.results.join(hs_obj.modules)
results = results.sort_values('Z', ascending=False)
results.to_csv("module_genes.csv")
module_scores = hs_obj.calculate_module_scores()
module_scores.to_csv("module_scores.csv")
```
