# Supplementary material for: Single-cell transcriptome sequencing reveals tumor heterogeneity in family neuroblastoma
Source: Front Immunol. 2023 Sep 18;14:1197773. doi: 10.3389/fimmu.2023.1197773 (PMC10543897; doi:10.3389/fimmu.2023.1197773)
Supplement: Supplementary file 5 [file DataSheet_2.pdf]

```
# Data analysis based R.
library(dplyr)
library(Seurat)
library(harmony)
library(viridis)
library(ggplot2)


#Input:
clustcol=c('#0067AA','#FF7F00','#00A23F','#FF1F1D','#A763AC','#B45B5D','#FF8AB6','#B6B800','#
01C1CC','#85D5F8')

data = readRDS('celltype_result.rds')


##################################################################################
#
## Seurat rds Visualization
# bar plot
cluster_num <- table( data@active.ident , data@meta.data[,'sample'])
cluster_num <- cluster_num[, colSums(cluster_num)>0]
ident<-rownames(cluster_num)
freq_table <- datap.table(x=cluster_num, margin=2)
labels <- as.character(rownames(freq_table))
rownames(freq_table)<-labels
rownames(cluster_num)<-labels
write.table(cluster_num,file='CellsPerCluster.xls',sep='\t',quote=F,row.names=T,col.names = NA)
write.table(freq_table,file='PercentPerCluster.xls',sep='\t',quote=F,row.names=T,col.names = NA)
freq_tab = melt(freq_table,by=0)
ggplot(freq_tab, aes(x=Var2, y=value, fill=Var1 ) ) + geom_bar(stat='identity',
position='stack',width=0.9) +
   scale_fill_manual(values=clustcol) + theme_classic() + labs(x='',y='',fill='') +
   theme(axis.text.x=element_text(angle=90,hjust=1), aspect.ratio=max(0.8,
15/length(levels(freq_tab$Var2)))) +
   guides(fill=guide_legend(ncol=1)) + scale_y_continuous(expand=c(0.003,0.003))

#Input: features
features = '' # genes to plot
FeaturePlot(data, features = features,cols = c("grey","red"))
DoHeatmap(data, features = features,label = F) + scale_fill_viridis()
DotPlot(data, features = features,cols = c("blue","red"))
VlnPlot(data, features = features, pt.size = 0, fill.by = 'ident', cols = clustcol, stack = T)
```

```
################################################################################
#
## Enrichment analysis.
library(KEGG.db)
library(org.Hs.eg.db)
library(clusterProfiler)
#Input: diffgene,GO_term,KEGG_term
genes <- read.table (diffgene,header = T)
data = bitr(genes$gene,
            fromType="SYMBOL",
            toType="ENTREZID",
            OrgDb="org.Hs.eg.db")
ego_ALL <- enrichGO(gene = data$ENTREZID,
                    OrgDb = org.Hs.eg.db,
                    ont = "ALL",
                    pAdjustMethod = "BH",
                    pvalueCutoff = 1,
                    qvalueCutoff = 1)
write.table(ego_ALL@result,"Enrichment_GO.xls",sep = "\t",col.names = T, row.names = F,quote
= F)
ego_ALL <- filter(ego_ALL, ego_ALL@result$Description %in% GO_term)
pdf("Enrichment_GO_dot.pdf")
dotplot(ego_ALL,title="Enrichment_GO_dot") + facet_grid(ONTOLOGY~.,scale='free',
space='free_y')
dev.off()
pdf("Enrichment_GO_bar.pdf")
barplt <- ggplot(obj@result,aes(x=Description,y=-log10(p.adjust),fill=ONTOLOGY)) +
geom_bar(stat='identity',width=0.85) + theme_classic() +

theme(axis.text.x=element_text(angle=90,hjust=1,vjust=0.5),aspect.ratio=1.6,axis.text.y=element
_text(size=12)) +
  scale_y_continuous(expand=c(0.01,0.01)) + coord_flip() +
scale_x_discrete(limits=rev(levels(obj@result$Description))) +
  labs(y=paste('-log10(',cutoff,')'),x='Pathways')
dev.off()
kk <- enrichKEGG(gene = data$ENTREZID,
                 organism = 'hsa',
                 pvalueCutoff = 1)
write.table(kk@result,"Enrichment_KEGG.xls",sep = "\t",col.names = T, row.names = F,quote = F)
kk <- filter(kk, kk@result$Description %in% KEGG_term)
pdf("Enrichment_KEGG_dot.pdf")
dotplot(kk,title="Enrichment_KEGG_dot")
dev.off()
```

```
pdf("Enrichment_KEGG_bar.pdf")
barplt <- ggplot(obj@result,aes(x=Description,y=-log10(get(cutoff)),fill=Count)) +
geom_bar(stat='identity',width=0.85) + theme_classic() +

theme(axis.text.x=element_text(angle=90,hjust=1,vjust=0.5),aspect.ratio=1.6,axis.text.y=element
_text(size=12)) +
  scale_y_continuous(expand=c(0.01,0.01)) + coord_flip() +
scale_x_discrete(limits=rev(levels(obj@result$Description))) +
  labs(y=paste('-log10(',cutoff,')'),x='Pathways') +
scale_fill_gradient(low='LightBlue',high='RoyalBlue')
dev.off()



################################################################################
#
## monocle
library(Seurat)
library(monocle)

pro = readRDS('celltype_result.rds')
pro <- FindVariableFeatures(pro, selection.method = "vst", nfeatures = 2000)
ordergene <- head(VariableFeatures(pro), use_gnum)
count <- GetAssayData(object = pro[["RNA"]], slot = "counts")
fdata <- data.frame(gene_short_name = row.names(count), row.names = row.names(count))
pdata <- pro@meta.data
my_cds <- newCellDataSet(count,
                         featureData = new("AnnotatedDataFrame", data =fdata),
                         phenoData = new("AnnotatedDataFrame", data = pdata),
                         lowerDetectionLimit = 0.5,
                         expressionFamily = negbinomial.size())
my_cds <- estimateSizeFactors(my_cds)
my_cds <- estimateDispersions(my_cds)
my_cds <- detectGenes(my_cds, min_expr = 0.1)
pData(my_cds)$UMI <- Matrix::colSums(exprs(my_cds))
my_cds <- setOrderingFilter(my_cds,ordergene)
my_cds <- reduceDimension(my_cds, reduction_method = 'DDRTree')
my_cds <- orderCells(my_cds)
pData(my_cds)$Pseudotime <- (pData(my_cds)$Pseudotime -
min(pData(my_cds)$Pseudotime))*10 / (max(pData(my_cds)$Pseudotime) -
min(pData(my_cds)$Pseudotime))
pData(my_cds)$Pseudotime <- round(pData(my_cds)$Pseudotime, 1)
my_pseudotime_de <- differentialGeneTest(my_cds, fullModelFormulaStr =
"~sm.ns(Pseudotime)", cores = 2)
```

```
gene_to_pseudotime <- my_pseudotime_de %>% arrange(qval) %>% head(30) %>% pull(gene_short_name) %>% as.character() %>% sort()

cell_size = 1
show_tree_argv = T
show_branch_points_argv = F
color_use = clustcol
ncol = 1
# gene expression plot
plot_genes_in_pseudotime(my_cds[gene_to_pseudotime,], ncol = 2, cell_size = cell_size, color_by = "cluster") +
  theme(legend.position = "top") +
  scale_color_manual(values = color_use, name = "Cluster") +
  theme(legend.position = 'right',
        axis.line=element_line(color="black",size=2),
        axis.text=element_text(size=12,face = "bold",color="black")) +
  guides(color = guide_legend(override.aes = list(size = 4), ncol = ncol))
# heatmap
ph <- plot_pseudotime_heatmap_sgr(my_cds[gene_to_pseudotime,], num_clusters = length(unique(pData(my_cds)$State)),
                                  cores = 1, show_rownames = TRUE, return_heatmap = TRUE)
#cluster
p1 <- plot_cell_trajectory(my_cds, color_by = "cluster", cell_size = cell_size, show_tree = show_tree_argv, show_branch_points = show_branch_points_argv) +
  scale_color_manual(values = color_use, name = "Cluster") +
  theme(legend.position='right', aspect.ratio=1,
        axis.line=element_line(color="black",size=2),
        axis.text=element_text(size=12,face = "bold",color="black")) +
  guides(color = guide_legend(override.aes = list(size = 4), ncol = ncol))
#Pseudotime
p2 <- plot_cell_trajectory(my_cds, color_by = "Pseudotime", cell_size = cell_size, show_tree = show_tree_argv, show_branch_points = show_branch_points_argv) +
  scale_color_viridis_c() +
  theme(legend.position='right', aspect.ratio=1,
        axis.line=element_line(color="black",size=2),
        axis.text=element_text(size=12,face = "bold",color="black"))
#state
p3 <- plot_cell_trajectory(my_cds, color_by = "State",cell_size = cell_size, show_tree = show_tree_argv, show_branch_points = show_branch_points_argv)   +
  theme(legend.position='right', aspect.ratio=1,
        axis.line=element_line(color="black",size=2),
        axis.text=element_text(size=12,face = "bold",color="black")) +
  guides(color = guide_legend(override.aes = list(size = 4), ncol=ncol))
#sample
```

```
p4 <- plot_cell_trajectory(my_cds, color_by = "sample",cell_size = cell_size, show_tree = show_tree_argv, show_branch_points = show_branch_points_argv) +
  scale_color_manual(values = samplecol_use) +
  theme(legend.position='right', aspect.ratio=1,
        axis.line=element_line(color="black",size=2),
        axis.text=element_text(size=12,face = "bold",color="black")) +
  guides(color = guide_legend(override.aes = list(size = 4), ncol=ncol))
# sample facet
fp2 <- plot_cell_trajectory(my_cds, color_by = "cluster", cell_size = cell_size, show_tree = show_tree_argv, show_branch_points = show_branch_points_argv) +
  scale_color_manual(values = color_use, name = "Cluster") +
  facet_wrap(~sample) +
  theme(legend.position='right', aspect.ratio=1,
        axis.line=element_line(color="black",size=2),
        axis.text=element_text(size=12,face = "bold",color="black")) +
  guides(color = guide_legend(override.aes = list(size = 4), ncol=ncol))

## BEAM
BEAM_res <- BEAM(my_cds, branch_point = 1, cores = 3)
BEAM_res <- BEAM_res[order(BEAM_res$qval),]
BEAM_res <- BEAM_res[,c("gene_short_name", "pval", "qval")]
num_cluster = 3
p <- plot_genes_branched_heatmap(my_cds[row.names(subset(BEAM_res, qval < 1e-4)),],
                                 branch_point = 1,
                                 num_clusters = num_cluster,
                                 cores = 3,
                                 use_gene_short_name = T,
                                 show_rownames = F,
                                 return_heatmap = T)



################################################################################
#
## Cell-cell interaction
library(CellChat)
library(ComplexHeatmap)
library(Seurat)
library(argparser)
library(igraph)
library(parallel)

rds.final<-readRDS(rdsfile)
rds.final@meta.data$cluster <- rds.final@active.ident
```

```
data.input<- rds.final@assays$RNA@data
identity = data.frame(group =rds.final@active.ident, row.names = names(rds.final@active.ident))
cellchat <- createCellChat(data.input)
cellchat <- updateCellChat(cellchat)
cellchat <- addMeta(cellchat, meta = identity, meta.name = "labels")
cellchat <- setIdent(cellchat, ident.use = "labels")
groupSize <- as.numeric(table(cellchat@idents))
signalPath = "All_signals"
CellChatDB <- CellChatDB.human
CellChatDB.use <- subsetDB(CellChatDB, search="All signals")
cellchatData@DB <- CellChatDB.use
cellchat <- subsetData(cellchatData)
cellchat <- identifyOverExpressedGenes(cellchat,thresh.p = 0.05)
cellchat <- identifyOverExpressedInteractions(cellchat)
cellchat <- projectData(cellchat, PPI.human)
cellchat <- computeCommunProb(cellchat, population.size=F, type="truncatedMean", trim = 0.1)
cellchat <- computeCommunProbPathway(cellchat)
cellchat <- aggregateNet(cellchat)
a<-cellchat@LR$LRsig
write.table(a,file="inter.xls",sep="\t",col.names=NA,row.names=T,quote=F)
cellchat@netP$pathways
write.table(cellchat@netP$pathways,file="netP.pathways.txt",sep="\t",col.names=F,row.names=F,
quote=F)
saveRDS(cellchat,file='cellchat.rds')
# plot
resultRDS="cellchat.rds"
Pathway="netP.pathways.txt"
plotOutdir="./"
cellchat<-readRDS(resultRDS)
netVisual_circle(cellchat@net$count, vertex.weight = groupSize, weight.scale = T, label.edge= F,
title.name = "Number of interactions")
netVisual_circle(cellchat@net$weight, vertex.weight = groupSize, weight.scale = T, label.edge= F,
title.name = "Interaction weights/strength")
pathways.show <- c("CXCL")
vertex.receiver = seq(1,8)
netVisual_aggregate(cellchat, signaling = pathways.show,    vertex.receiver = vertex.receiver)
netVisual_aggregate(cellchat, signaling = pathways.show, layout = "circle")
netVisual_heatmap(cellchat, signaling = pathways.show, color.heatmap = "Reds")
netAnalysis_contribution(cellchat, signaling = pathways.show)
netVisual_bubble(cellchat, sources.use = 4, targets.use = c(5:11), remove.isolate = FALSE)
# cellchat comparison of the two groups
cellchat1 <- readRDS(rds1)
cellchat2 <- readRDS(rds2)
cc.list <- list()
```

```
cc.list[["g1"]] <- cellchat1
cc.list[["g2"]] <- cellchat2
cc.list$g1@DB <- CellChatDB.human ; cc.list$g2@DB <- CellChatDB.human
cc.list <- lapply(cc.list, function(x) subsetData(x))
cc.list <- lapply(cc.list, function(x) updateCellChat(x))
cc.list <- lapply(cc.list, function(x) identifyOverExpressedGenes(x))
cc.list <- lapply(cc.list, function(x) identifyOverExpressedInteractions(x))
cc.list <- lapply(cc.list, function(x) computeCommunProb(x, population.size = F, type = "truncatedMean", trim = 0.1))
cc.list <- lapply(cc.list, function(x) filterCommunication(x, min.cells = 10))
cc.list <- lapply(cc.list, function(x) computeCommunProbPathway(x))
cc.list <- lapply(cc.list, function(x) aggregateNet(x))
object.list = cc.list
names(object.list)<-grouplist
cellchat <- mergeCellChat(object.list, add.names = names(object.list))
# plot
gg1 <- compareInteractions(cellchat, show.legend = F, group = c(1,2))
gg2 <- compareInteractions(cellchat, show.legend = F, group = c(1,2), measure = "weight")
gg1 + gg2

netVisual_diffInteraction(cellchat, weight.scale = T)
netVisual_diffInteraction(cellchat, weight.scale = T, measure = "weight")

gg1 <- netVisual_heatmap(cellchat)
gg2 <- netVisual_heatmap(cellchat, measure = "weight")
gg1 + gg2

weight.max <- getMaxWeight(object.list, attribute = c("idents","count"))
par(mfrow = c(1,2), xpd=TRUE)
for (i in 1:length(object.list)) {
  netVisual_circle(object.list[[i]]@net$count, weight.scale = T, label.edge= F, edge.weight.max = weight.max[2], edge.width.max = 12, title.name = paste0("Number of interactions - ", names(object.list)[i]))
}

num.link <- sapply(object.list, function(x) {rowSums(x@net$count) + colSums(x@net$count)-diag(x@net$count)})
weight.MinMax <- c(min(num.link), max(num.link)) # control the dot size in the different datasets
gg <- list()
for (i in 1:length(object.list)) {
  gg[[i]] <- netAnalysis_signalingRole_scatter(object.list[[i]], title = names(object.list)[i], weight.MinMax = weight.MinMax)
}
patchwork::wrap_plots(plots = gg)
```

```
gg1 <- rankNet(cellchat, mode = "comparison",stacked = T, do.stat = TRUE)
gg2 <- rankNet(cellchat, mode = "comparison", stacked = F, do.stat = TRUE)
gg1+gg2

gg1 <- rankNet(cellchat, mode = "comparison", stacked = T, do.stat = TRUE)
gg2 <- rankNet(cellchat, mode = "comparison", stacked = F, do.stat = TRUE)
gg1 + gg2

i = 1
pathway.union <- union(object.list[[i]]@netP$pathways, object.list[[i+1]]@netP$pathways)
ht1 = netAnalysis_signalingRole_heatmap(object.list[[i]], pattern = "outgoing", signaling = pathway.union, title = names(object.list)[i], width = length(celltype)*0.34, height = length(pathway.union)*0.23)
ht2 = netAnalysis_signalingRole_heatmap(object.list[[i+1]], pattern = "outgoing", signaling = pathway.union, title = names(object.list)[i+1], width = length(celltype)*0.34, height = length(pathway.union)*0.23)
draw(ht1 + ht2, ht_gap = unit(0.5, "cm"))
ht1 = netAnalysis_signalingRole_heatmap(object.list[[i]], pattern = "incoming", signaling = pathway.union, title = names(object.list)[i], width = length(celltype)*0.34, height = length(pathway.union)*0.23, color.heatmap = "GnBu")
ht2 = netAnalysis_signalingRole_heatmap(object.list[[i+1]], pattern = "incoming", signaling = pathway.union, title = names(object.list)[i+1], width = length(celltype)*0.34, height = length(pathway.union)*0.23, color.heatmap = "GnBu")
draw(ht1 + ht2, ht_gap = unit(0.5, "cm"))

celltype1<-apply(cellchat1@net$count,1,sum)
celltype2<-apply(cellchat2@net$count,1,sum)
celltype<-intersect(names(celltype1[celltype1>0]), names(celltype2[celltype2>0]))

netVisual_bubble(cellchat, sources.use = 1, targets.use=c(2:15), comparison = c(1, 2), angle.x = 45)
netVisual_bubble(cellchat, sources.use = c(2:15), targets.use=1, comparison = c(1, 2), angle.x = 45)
```
